# Corticosteroids showed more efficacy in treating hospitalized patients with COVID-19 than standard care but the effect is minimal: A systematic review and meta-analysis

**DOI:** 10.3389/fpubh.2022.847695

**Published:** 2022-07-22

**Authors:** Jixin Liu, Jing Dong, Yage Yu, Xinna Yang, Juan Shu, Hairong Bao

**Affiliations:** ^1^Medical Service Center, Gansu Provincial Hospital, Lanzhou, China; ^2^Geriatric Respiratory Department, First Hospital of Lanzhou University, Lanzhou, China

**Keywords:** COVID-19, corticosteroids, clinical benefit, systematic review, meta-analysis

## Abstract

**Background:**

During the ongoing coronavirus disease 2019 (COVID-19) pandemic, the use of corticosteroids for COVID-19 has ignited worldwide debate. Previous systematic reviews, including randomized controlled trials (RCTs) and retrospective observational studies, found that corticosteroids have beneficial effects in treating COVID-19.

**Aim:**

This systematic review and meta-analysis only included RCTs to assess the effectiveness and safety of corticosteroids in hospitalized patients with COVID-19.

**Methods:**

Comprehensive research strategies (PubMed, Embase, MEDLINE, and Coherence Library) were used to search for RCTs from December 2019 to January 2021.

**Results:**

Five RCTs were included with 7,235 patients, of which 2,508 patients were receiving corticosteroid treatments (dexamethasone or methylprednisolone), and 4,727 received standard care. The primary outcome was mortality within 28 days. The use of corticosteroids decreased the 28-day mortality of patients with COVID-19, but the findings were not statistically significant (RR, 0.91; 95% CI, 0.78–1.06, *p* = 0.24). The secondary outcome was the duration of hospitalization; no differences were found between the corticosteroid and standard care groups. However, corticosteroids were associated with a higher hospital discharge rate than standard treatment, but the result was not statistically significant (RR, 1.36; 95% CI, 0.95–1.96, *p* = 0.09).

**Conclusions:**

The results suggest that corticosteroids are comparable to standard care in terms of safety in treating COVID-19. Corticosteroids showed greater efficacy than standard care; however, the effect was minimal.

## Introduction

Since December 2019, several hospitals in Wuhan City, Hubei Province, have reported multiple cases of unexplained pneumonia with a history of travel to the South China seafood industry, believed to be an acute respiratory infectious disease triggered by the 2019 new coronavirus outbreak, also known as “New Coronavirus Pneumonia” (1–3).

According to existing case data, new coronavirus pneumonia mainly manifests as fever, dry cough, and fatigue (4, 5). A small number of patients present with nasal congestion, runny nose, diarrhea, and other upper respiratory and digestive tract symptoms. Severe cases often have difficulty breathing after 1 week, and rapidly progress to acute respiratory distress syndrome, septic shock, difficulty correcting metabolic acidosis, coagulation dysfunction, and multiple organ failure (6–8).

Corticosteroids are commonly used in the treatment of former coronavirus infections such as severe acute respiratory syndrome (SARS) and Middle East respiratory syndrome (MERS) and are now one of the treatment choices for new coronavirus infections (2019-nCoV) (9, 10). However, evidence for its effectiveness is inconclusive. The preliminary results of the RECOVERY trial show that the use of dexamethasone can reduce the 28-day mortality of coronavirus disease 2019 (COVID-19) patients, but only among patients who receive invasive mechanical ventilation (11). Systematic reviews, including randomized controlled trials (RCTs) and observational studies, have shown that corticosteroid use has a beneficial effect on reducing the mortality rate among severe patients with COVID-19 (12). This systematic review and meta-analysis included only RCTs to evaluate the effectiveness and safety of corticosteroids in the treatment of COVID-19.

## Methods

### Data sources

**Figure 1:**
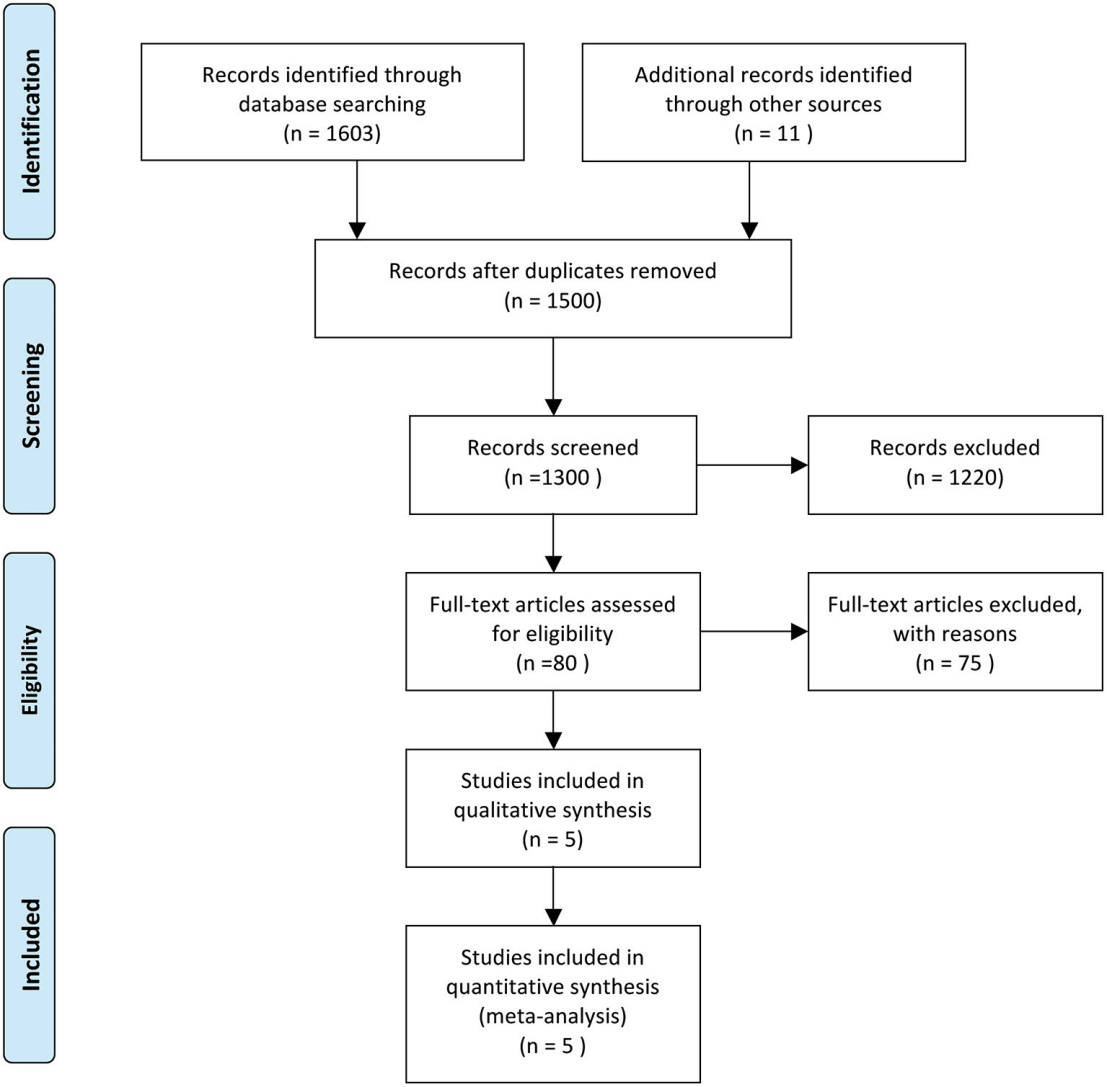
Literature searching followed PRISMA 2015. From Moher et al. (13).

We conducted a systematic review and meta-analysis based on the preferred reporting items for systematic review and meta-analysis protocols (PRISMA-P) 2015 (Figure 1) (14). Two reviewers (JL and JD) independently searched PubMed, Embase, and Cochrane Collaborative Controlled Trial Centers from Dec 2019 to Feb 2021. The third author resolved all the disputes (HRB). The following headings are used for searches: “SARS-CoV-2,” “Glucocorticoids,” “Dexamethasone,” “Methylprednisolone,” and “Clinical Outcomes.”

### Selection criteria and data extraction

The meta-analysis used the following inclusion criteria: (1) the study design was an RCT (2) the study directly compared corticosteroids with placebo or standard care, (3) the study included patients who were older than 18 years, had confirmed or suspected COVID-19, (4) the study reported mortality at 28 days. The exclusion criteria included (1) ongoing or incomplete studies, (2) retrospective observational studies, (3) patients included in the studies were under 18 years old or pregnant women, (4) patients who were non-hospitalized, and (5) mortality was not reported in the Results section.

### Primary and secondary outcomes and definitions

The primary outcome was all-cause mortality at 28 days and the need for oxygen assistance (nasal cannula, mask oxygen, reservoir mask, non-invasive ventilation (NIV), invasive mechanical ventilation (IMV), and orotracheal intubation). The periods of hospitalization and hospital discharge were secondary results. The other outcomes included clinical improvements (defined by the studies as improved dyspnea, no fever for 72 h, Borg score >3, tolerated oral regimen, standard urinary output, free from oxygen support), early mortality (on days 7 and 14), and any adverse events.

### Quality assessment

**Figure 2:**
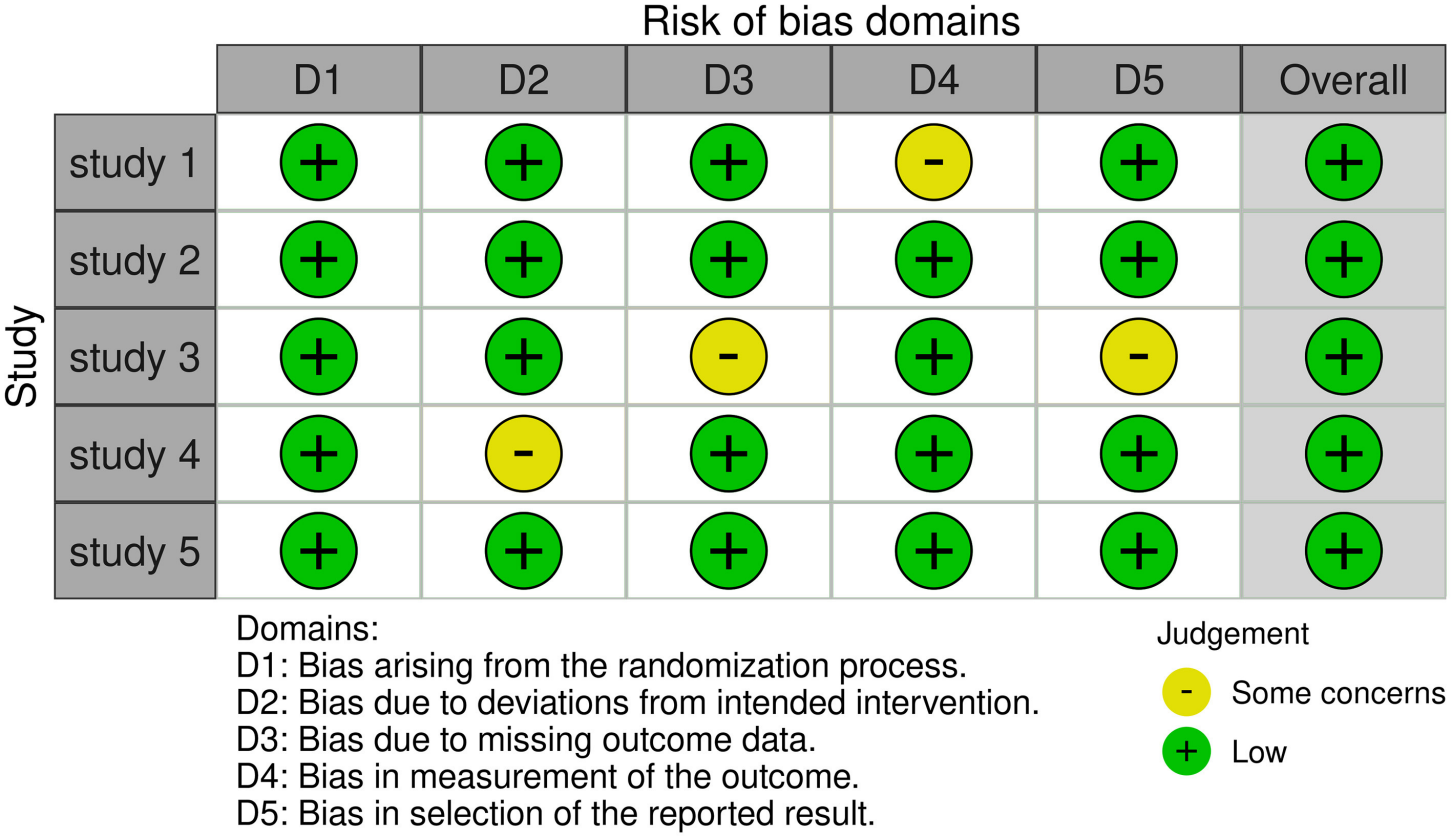
Quality assessment of included RCTs.

Two authors separately assessed the quality of the included RCTs based on version 2 of the Cochrane Risk of Bias Assessment Tool (15) and rated them as random sequence generation, allocation concealment, patient and healthcare provider blindness, data collector blinding, outcome assessor blinding, and insufficient outcome data, as well as some other bias (Figure 2).

### Statistical analysis

For dichotomous results, we used the Mantel–Haenszel method to measure overview risk ratios (RRs) and 95 percent confidence intervals (CIs), and we used a random-effects model to account for between-study heterogeneity. We used Cochran's Q statistics and I2 test to assess the heterogeneity of the included RCTs.

## Results

### Included studies characteristics

After a comprehensive literature search, a total of five RCTs were included in the meta-analysis. Two studies compared methylprednisolone plus standard care with standard care alone (16, 17), including a total of 461 patients, of which 34 received methylprednisolone intravenously at 250 mg/day for 3 days plus standard care (16), 194 received methylprednisolone intravenously at a dose of 0.5 mg/kg twice daily for five days plus standard care (17), and 233 received standard treatment only. Three studies compare dexamethasone plus standard care with standard care alone (11, 18, 19). In total, 6,774 patients were included. Among them, 176 patients received dexamethasone 20 mg/day for 5 days, followed by 10 mg/day for 5 days, with additional standard care (18, 19). A total of 2,104 patients received oral or intravenous dexamethasone 6 mg/day for 10 days plus standard care (11), and the remaining 4,494 patients received standard care only (Table 1).

**Table 1:** Included randomized controlled clinical trials characteristics.

**Studies**	**Design+** **Locations**	**Study period**	**Treatment**	**Control**	**Total no**.	**Primary outcome**	**Treatment**	**Control**	***p*** **-value**
Tamazini et al. (19)	Multicentered; 41 ICUs in Brazil	April 17-Juen 23, 2020	20 mg of dexamethasone/day for 5 days; 10 mg of dexa. For 5 days + Usual care	Usual care	*N =* 299	Ventilator-free during the first 28 days	54/151	43/148	0.002
Group et al. (11)	RECOVERY trail 176 NHSO in the UK	March 19-June 8, 2020	Oral or intravenous dexa. At a dose of 6 mg/day for 10 days	Usual care	*N =* 6,425	28-day mortality	482/2104	1110/4321	<0.001
Jamaati et al. (18)	Iran		Intravenous dexa. At a dose of 20 mg/day for 5 days 10 mg/day for 5days	Usual care	*N =* 50	Non-invasive ventilation	9/9	10/10	0.500
Edalatifard et al. (16)	Iran	April 20-June 20, 2020	Intravenous injection methylprednisolone 250 mg/day for 3 days	Usual care	*N =* 68	Recovery	32/34	16/34	<0.001
Jeronimo et al. (17)	Western Brazilian Amazon	April 18-June 16, 2020	Intravenous MP (0.5 mg/kg)	Placebo	*N =* 393	28-day mortality	72/194	76/199	0.629

### Primary outcome

**Figure 3:**
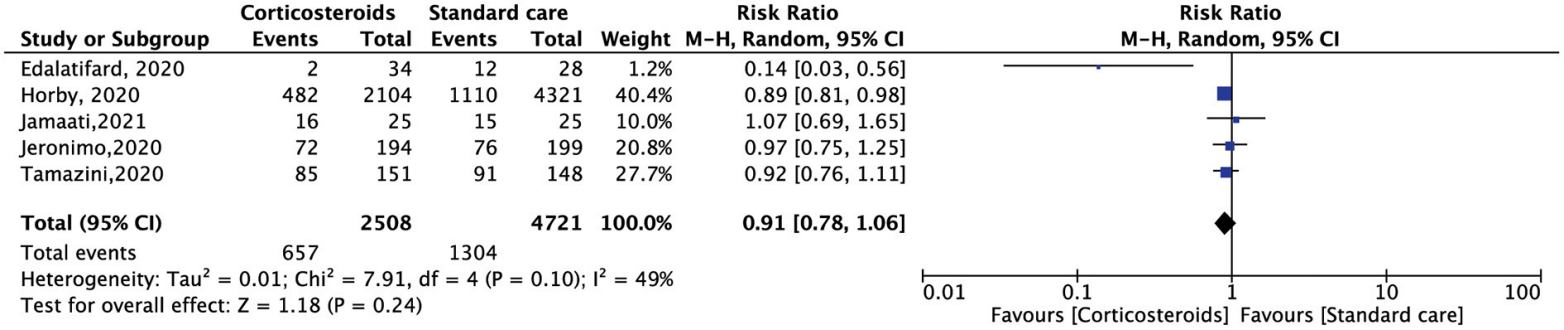
Forest plot of comparison: 28-day mortality.

**Figure 4:**
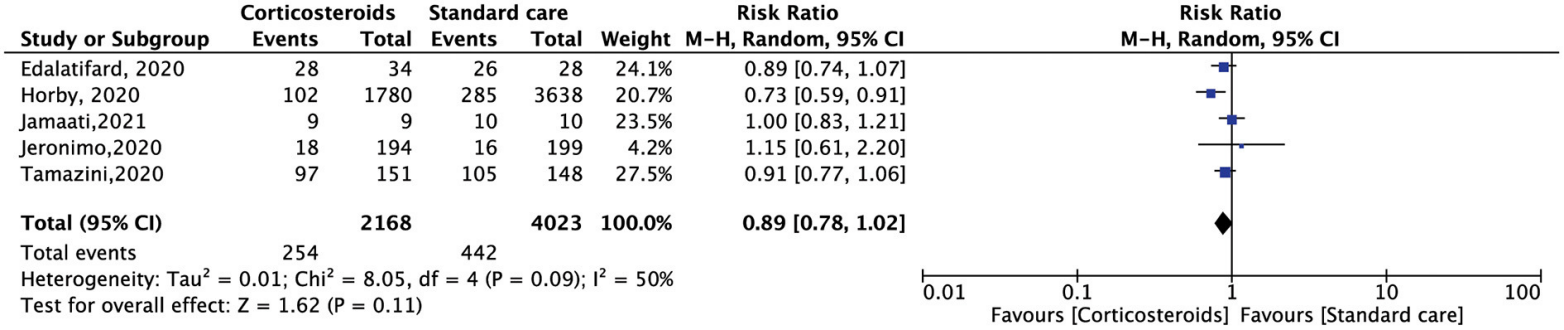
Forest plot of comparison: demand for oxygen support.

All the included RCTs reported a mortality rate within 28 days, involving 7,229 patients. Compared with standard care alone, corticosteroid use reduced the 28-day mortality of hospitalized patients with COVID-19, but the results were not statistically significant (RR, 0.91; 95% CI: 0.78–1.06, *p* = 0.24) (Figure 3). The need for oxygen support also showed the same results. Five RCTs included 6,191 patients. Compared with standard care alone, corticosteroids reduced the demand for oxygen support, but the result was not statistically significant (RR, 0.89; 95% CI: 0.78–1.02, *p* = 0.11) (Figure 4).

### Secondary outcome

**Figure 5:**
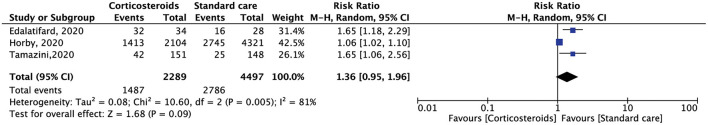
Forest plot of comparison: hospital discharge rate.

Four RCTs reported on the duration of hospitalization. Compared with standard care alone, the use of corticosteroids does not reduce the length of hospital stay, whereas one RCT showed that corticosteroids reduce the length of ICU stay (18). Three RTCs reported the results of the discharge rate of 6,786 patients. The results showed that compared with standard care, corticosteroids were associated with a greater hospital discharge rate, but this was not statistically significant (RR, 1.36; 95% CI: 0.95–1.96, *p* = 0.09) (Figure 5).

### Other outcomes

An RCT reported that, compared with standard care, corticosteroids did not significantly benefit clinical improvements for treating hospitalized patients with COVID-19 (18). One RCT addressed the early mortality rate, and the results showed no significant difference between corticosteroids and standard care (17). Two RCTs showed that the use of corticosteroids did not result in any adverse events compared to standard care (11, 16).

## Discussion

The results of this systematic review and meta-analysis were different from those of previous studies. First, compared with standard care, corticosteroids do not reduce the mortality of hospitalized patients with COVID-19 within 28 days. Second, compared to the usual care group, the need for oxygen therapy was not significantly different in the corticosteroid group. However, the use of corticosteroids has significantly increased the discharge rate of patients with COVID-19. For other outcomes, including clinical benefit, early mortality, and adverse events, corticosteroiduse was not significantly different from standard care.

A previous prospective meta-analysis of seven randomized clinical trials was conducted. Patients with severe new coronary pneumonia treated with systemic corticosteroids (dexamethasone, hydrocortisone, or methylprednisolone) had a 28-day all-cause death rate that decreased by 20% compared to patients undergoing standard treatment or placebo (20).

Different results were found in this systematic review and meta-analysis and previous review articles published online by The Lancet. Researchers from the University of Edinburgh have found that corticosteroid therapy is not practical for patients with new coronary pneumonia (21). Similar treatments are not recommended for lung damage or shock caused by the new coronavirus (21, 22). Corticosteroids have been widely used to treat SARS (23, 24). However, the clinical guidelines issued by the World Health Organization on January 28, 2020, recommend that unless otherwise specified, corticosteroids should not be used for people who are not severely infected with the new coronavirus (25). Patients with new coronary pneumonia develop acute lung injury and acute respiratory distress syndrome caused by the host immune response (5). Corticosteroids can suppress lung inflammation, suppress immune response, and prevent it from clearing pathogens (21). This article compares the clinical data of corticosteroid treatment for the new coronavirus with other similar diseases. No clinical data have shown that corticosteroids are beneficial for treating of respiratory infections caused by viruses, such as SARS and MERS (21). Similar results were found in the RECOVERY trial, in which there was no indication that dexamethasone offered any advantage to patients who did not provide respiratory assistance at the time of randomization (11).

Although this meta-analysis and previous studies have shown that corticosteroids have no significant effect on the treatment of patients with COVID-19, however, a previous systematic review and RCTs found that compared with dexamethasone, methylprednisolone may have a better effect on the treatment of hospitalized patients with COVID-19 in terms of a lower mortality rate (9, 26). However, because of the few included studies, no firm conclusions could be drawn from these studies.

Corticosteroids are not used in treatment protocol to target virus but to attenuate the consequences of the infection. Several *in vitro* studies have shown that the dexamethasone and prednisolone are not directly acting as a potent antiviral, but used to minimize the cytokine storm in severe cases (27). Therefore, their impact may be not clear in RCTs including mild-to-moderate COVID-19 cases.

This meta-analysis has several limitations: (1) because there are few RCTs comparing corticosteroids and standard care, there are relatively few studies included in the meta-analysis, and there may be bias; (2) there are differences between each study, including the dose of the medication, the time of treatment, and follow-up duration; and (3) regarding the results of the duration of hospitalization, we did not find enough data in the included studies for meta-analysis.

## Conclusion

Our meta-analysis indicates that, in contrast to previous studies, among hospitalized patients with COVID-19, the use of corticosteroids increases the hospital discharge rate. However, other clinical outcomes showed no differences with standard care, including 28-day mortality, need for oxygen support, duration of hospitalization, clinical improvements, early mortality, and adverse events.

## Data availability statement

The original contributions presented in the study are included in the article/supplementary material, further inquiries can be directed to the corresponding author/s.

## Author contributions

JL and JD designed the study and drafted the manuscript. YY, XY, and JS were involved in literature selection and data analysis. JL and HB revised the manuscript accordingly. HB was responsible for this project and commented on the manuscript accordingly. All authors have read and approved the final version of the manuscript.

## Funding

The study was financially supported by the Key R&D Plan, Gansu Science and Technology Program (20YF2FA013) and the Innovation Base and Talent Plan, Gansu Science and Technology Program (20JR10FA666).

## Conflict of interest

The authors declare that the research was conducted in the absence of any commercial or financial relationships that could be construed as a potential conflict of interest.

## Publisher's Note

All claims expressed in this article are solely those of the authors and do not necessarily represent those of their affiliated organizations, or those of the publisher, the editors and the reviewers. Any product that may be evaluated in this article, or claim that may be made by its manufacturer, is not guaranteed or endorsed by the publisher.
